# Cervical cancer screening service utilization and associated factors among age-eligible women in Jimma town using health belief model, South West Ethiopia

**DOI:** 10.1186/s12905-019-0826-y

**Published:** 2019-10-28

**Authors:** Tadesse Nigussie, Bitiya Admassu, Aderajew Nigussie

**Affiliations:** Mizan-Tepi University, Mizan Aman, Ethiopia

**Keywords:** Cervical cancer, Cervical cancer screening, Health belief model, Jimma town

## Abstract

**Background:**

Cervical cancer is the second most common gynecologic cancer affecting the lives of women. It causes hundreds of thousands of death among women annually worldwide. When a woman is screened for cervical cancer at least once in her life between the ages of 30 and 40, the risk of getting cervical cancer can be decreased by 25–36%. Despite this advantage, the coverage of cervical cancer screening is limited in low and middle-income countries including Ethiopia.

**Objective:**

To assess cervical cancer screening service utilization and associated factors among age-eligible women in Jimma town, South West Ethiopia, 2017.

**Methods:**

Community based cross-sectional study was used. Seven hundred thirty-seven women were selected using systematic random sampling. Data were collected using a structured interview administered questionnaire. Data were collected on socio-demographic, reproductive factors, knowledge of cervical cancer as well as constructs of Health belief model and practice related variables. Logistic regression analysis was performed, and variables with a *p*-value of less than 0.05 in the multivariable analysis were taken as statistically significant predictors of cervical cancer screening service utilization.

**Results:**

Of the 737 women, only 15.5% were screened for cervical cancer. The independent predictors of cervical cancer screening utilization were: being government employee [AOR = 3.00, 95% CI: 1.49–6.01], knowing someone who has ever screened [AOR = 3.61, 95% CI: 2.07–6.29], having history of gynecologic examination for any reason (having previous examination that expose women genitalia for physician like examination during child birth, abortion procedure and examination for STI) [AOR =2.84, 95% CI: 1.48–5.45], not preferring gender of physician for gynecological examination [AOR = 3.57, 95% CI: 1.98–6.45], getting advice from health care providers [AOR = 4.45, 95% CI: 2.57–7.70], having good knowledge of cervical cancer screening [AOR = 3.46, 95% CI: 1.47–8.21] and having perceived susceptible for cervical cancer [AOR = 3.03, 95% CI: 1.64–5.56].

**Conclusions:**

The utilization of cervical cancer screening services was low in Jimma town. Strengthening the screening service is important through raising the awareness of the community towards cervical cancer and screening services.

## Background

Cervical cancer is the second most common cancer among women worldwide. It contributes to the death of 266,000 and new cases of 528,000 in women annually [1]. The burden of the disease is high in low and middle-income countries (LMICs). They share about 85% of morbidity and 87% of death due to cervical cancer. The incidence of cervical cancer in sub-Saharan Africa is 35 per 100,000 women, and 23 death occur per 100,000 women every year [1]. In Ethiopia, cervical cancer was the second leading cause of death next to breast cancer among women aged 15–44 years in 2012. In the same year, 7095 new cases were diagnosed and 4732 deaths occurred due to cervical cancer. It contributed about 11% of all cases of cancer. The population at risk for cervical cancer was 29.4 million in 2012 [2]. A recent report from Ethiopia revealed that the trends of cervical cancer were increasing over the last 16 years in the country [3].

Poor and medically underserved population are highly affected by cervical cancer. These include sub-Saharan Africa, some parts of Latin America and the Caribbean [4]. In 2012 WHO estimated that within the following 10 years about 3.65 billion US dollars is required to combat cervical cancer through vaccination of girls, screening services and treatment of cervical cancer in LMIC [5]. In LMICs, there is limited resources to implement preventive services for cervical cancer. Besides, a screening program is not implemented in all health facility though there is supportive health policy. This causes disparity in mortality and morbidity from cervical cancer between high income countries (HICs) and LMICs [6, 7]. The study conducted in Addis Ababa indicated that lack of trained health professionals, budget, and low service coverage are barriers of cervical cancer screening utilization in Ethiopia [8].

The cervical cancer screening coverage is very low in Ethiopia. Less than 1 % of eligible women were screened in the country [9]. Also, only a small proportion of women have awareness concerning cervical cancer and cervical cancer screening. For instance, a study from Jimma University Medical Center showed that out of 60 patients who developed invasive cervical cancer, only 2(3.3%) of them had a history of cervical cancer screening and only 7(11.7%) of them heard about cervical cancer [10]. Another study from North West Ethiopia indicated a significant number of women have low knowledge about cervical cancer [11].

To increase the coverage and awareness of cervical cancer screening, the Ethiopian government made some efforts. For instance, the cancer registry was finalized in Addis Ababa and regional states (including Jimma) in 2015 to intervene in the consequences of cervical cancer, to promote cancer surveillance, to register and to research cervical cancer. As a result, a total of 22,818 women aged 30–49 undergone cervical cancer screening in 2015 [12]. Study from Mekele zone, Ethiopia showed that the proportion of screened women reached 19.8% in the 2015 year [13]. Even if, it is not at its expected level, cervical cancer screening service utilization is increasing in Ethiopia. Though there are very limited studies conducted in the country to assess the utilization of cervical cancer screening in Ethiopia [11, 13, 14]. Thus, this study identified factors associated with cervical cancer screening service utilization among eligible women (30–49 years) using the Health Belief Model (HBM).

HBM was originated in the 1950s to predict a person’s attitudes and actions (behaviors) regarding health issues and has been refined over the years [15]. The HBM suggests that people will take action to prevent, to screen for, or to control conditions of ill health if they regard themselves as susceptible to the condition, if they believe it could have potentially serious consequences, if they believe that a course of action available to them would be beneficial in reducing either their susceptibility to or the severity of the condition, and if they believe that the anticipated barriers to taking the action are outweighed by its benefits [16, 17]. This model has been applied in several research studies to understand the practice of cervical cancer screening [13, 18–20].

## Methods

### Study design and setting

Community based cross-sectional study was conducted in Jimma town among women of 30–49 years of age. Jimma town is the capital of Jimma zone which is 352 Km far from Addis Ababa to south-west Ethiopia. According to Jimma town health office in 2017, there were about 26,971 women of 30–49 years in Jimma town [21]. There are two public hospitals, four health centers and more than 15 private clinics providing health services in Jimma town. From these health facilities, two hospitals and two Non-Governmental Organization (NGO) clinics provide cervical cancer screening by using visual inspection of the cervix with acetic acid (VIA) [21]. The cervical cancer screening services were free of charge in government health facilities and women can utilize services through self-referral, referral by health extension workers and by referral of other health care professionals (nurses, midwifes, doctors and public health professionals). Also government is transmitting information through local and national media information regarding cervical cancer. The study was conducted from March 20 to April 15, 2017.

### Population

All age-eligible women (30–49 years) according to Ethiopian cervical cancer screening guideline [22] in Jimma town were source population (study base). Study populations were randomly selected age-eligible women for cervical cancer screening. Women who had lived at least 6 months in the town were included. Whereas women who were severely ill and unable to give responses during the data collection period were excluded from the study.

### Sample size and sampling procedure

Sample size was determined by using single population proportion formula by considering the following assumptions: *p* = 19.8% the prevalence of cervical cancer screening from a study conducted in Mekele Zone, Northern Ethiopia [13], d = 3% the margin of error, Z_α/2_ = 1.96 at 95% confidence of certainty. Thus, n = [(Z_α/2_)^2^ *p (1-p)]/ d^2^ = 678, considering 10% non-response rate = 68 and the final total sample size was 746.

Six Kebele (the smallest administrative unit in Ethiopia next to district) were randomly selected from a total of 17 kebeles of Jimma town for the study. Then, the sample was allocated proportionally to each Kebele based on the households of each kebeles. A systematic random sampling technique was used in selecting study participants. The sample interval (k = 20) was determined based on the total number household in kebeles. Further, for a household with more than one eligible woman, one woman was randomly selected by using the lottery method.

### Data collection tool

The data were collected using a structured interview administered questionnaire which was adapted from related literature [23–25]. It has five parts: **socio-demographic** characteristics (age, religion, educational status, marital status, family average monthly income and current occupation), reproductive characteristics (age of first sexual intercourse, parity, use of modern contraceptive, history of STD, history of HIV test and self-reported HIV sero-status), knowledge of cervical cancer and screening (it was measured by 15 questions having choice of yes, no and I don’t know), practice assessment (8 questions were used for screening and related practices), construct of health belief model (perceived susceptibility 3, perceived severity 6, perceived benefit 5 and perceived barriers 10 questions) (Table 3). Cronbach’s alpha coefficients were checked to test the internal consistency and reliability of the HBM constructs. Perceived susceptibility was measured by three items with Cronbach alpha of 0.83, perceived severity was measured with six items with Cronbach alpha of 0.84 while perceived benefit was measured with five items which gave Cronbach alpha of 0.87 and perceived barriers were measured with 10 items with Cronbach alpha of 0.92.

The questionnaire was translated from English to local language Afan Oromo and back to English by an independent person to assure its consistency. The questionnaire was pre-tested in Agaro town which is 40 km from Jimma town on 5% of the total sample size. The wording and sequence of the questionnaire were corrected based on pretest result. Two days of training was given for data collectors on data collection tools, interviewing techniques, maintaining the privacy and confidentiality of the respondents.

Data were collected by 10 BSc nurses. Supervision was made by three supervisors who were BSc in public health. Before doing the actual analysis, the data were checked for completeness, clarity, and consistency by the principal investigator.

### Operational definition and measurements

- ❖ **Knowledge about cervical cancer and screening:** 15 knowledge questions were used and correct answers were categorized as 1 while incorrect answers were categorized as 0. The total point scored was 15 and the minimum was 0. Then, the scores with their respective knowledge levels were 12–15 good knowledge, 8–11 satisfactory knowledge, 0–7 poor knowledge [26].
- ❖ **Perceived susceptibility for cervical cancer:** Beliefs about the chances of experiencing a risk or getting cervical cancer.
- ❖ **Perceived severity of cervical cancer:** Beliefs about how serious and sequel of cervical cancer.
- ❖ **The perceived benefit of undergoing cervical cancer screening:** Beliefs in the efficacy of the advised action to reduce the risk or seriousness of cervical cancer.
- ❖ **Perceived barriers for undergoing cervical cancer screening:** Beliefs about the tangible and psychological factors to undergo cervical cancer screening.
- ❖ Perceived susceptibility, severity, benefit and barriers were assessed using the Likert Scale (1 = strongly disagree, 2 = Disagree, 3 = Neutral, 4 = Agree, 5 = strongly agree). Mean scores were computed and dichotomized into high/positive and low/negative.
- ❖ ***Practice assessment:*** The practice was assessed by asking the respondent’s action towards screening for premalignant cervical lesions in the past 5 years. Those who ever screened within the past 5 years were regarded as having practice and those who never screened were regarded as having no screening practice.
- ❖ **Kebele:** the smallest administrative unit in Ethiopia next to district.
- ❖ **Gynecologic examination:** Any history of examination that make women to expose her genitalia to health professional like examinations for abortion, delivery and sexual transmitted disease examination.

### Data processing and analysis

The data were entered into Epi-data manager version 4.0.2.101 and exported to SPSS version 21 statistical packages for analysis. Frequencies and proportions were done for different variables. Bivariate analyses were performed to select variables for multivariable analysis. Hence variables with a *p*-value < 0.1 in the bivariate analyses were taken as candidates for multivariable analysis. Finally, multivariable logistic regression analyses was performed to control for the possible confounding effect of the selected variables and variables with a p-value of less than 0.05 were taken as statistically significant determinants for cervical cancer screening utilization.

## Result

### Socio demographic characteristics

A total of 737 women were interviewed from six selected kebeles making a response rate of 98.8%. The mean (± SD) age of the respondents was 36.6 (±5.3) years. About 304(41.2%) were Muslims by religion. Most of the respondents, 610(82.8%) were married. Two hundred fifteen (29.2%) respondents were attended tertiary education. Concerning occupation, 255(34.6%) were housewives (Table 1).

**Table 1:** Socio-demographic characteristics of women of age-eligible for cervical cancer screening (30–49 years) in Jimma town, south west Ethiopia (*n* = 737) June, 2017

Variable	N (%)
Age, Years	
30–39	508(68.9)
40–49	229(31.1)
Religion	
Muslim	304(41.2)
Orthodox	261(35.4)
Protestant	167(22.7)
Catholic	5(0.7)
Marital status	
Married	610(82.8)
Widowed	56(7.6)
Divorced	37(5.0)
Single	34(4.6)
Educational status	
No education	166(22.5)
Primary education (1–8)	196(26.6)
Secondary education (9–12)	160(21.7)
College and above	215(29.2)
Occupational status	
House wife	255(34.6)
Government employee	199(27.0)
Merchant	206(28.0)
Daily laborer	76(10.4)
Income status	
< 900	93(12.6)
901–1600	148(20.1)
1601–2700	110(14.9)
> 2700	386(52.4)

### Reproductive characteristics

Five hundred seventy-one (77.8%) of respondents had started sexual intercourse before the age of 20 years. Most of them, 661 (90.5%) had a history of childbirth. Out of these, 425(57.7%) had three or more children. Regarding contraceptive use, the majority of the respondents 587 (79.6%) had a history of modern contraceptive use (Table 2).

**Table 2:** Reproductive Characteristics of age-eligible women for cervical cancer screening (30–49 years) in Jimma town, south west Ethiopia (n = 737) June, 2017

Variables	N (%)
Age of first sexual intercourse, Years	
< 20	571(77.8)
≥ 20	163(22.2)
Parity	
< 3 children	236(35.7)
≥ 3 children	425(64.3)
Use of modern contraceptive	
Yes	587(79.6)
No	150(20.4)
History of STD	
Yes	108(14.7)
No	629(85.3)
History of HIV test	
Yes	657(89.1)
No	80(10.9)
Self-reported HIV sero status	
Positive	25(3.8)
Negative	632(96.2)

### Knowledge about cervical cancer

**Fig. 1:**
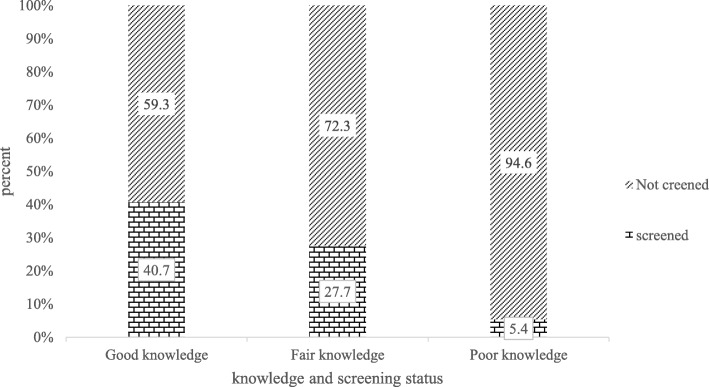
Knowledge of cervical cancer and screening service utilization among 30–49 years of age in Jimma town, south west Ethiopia, June, 2017

**Fig. 2:**
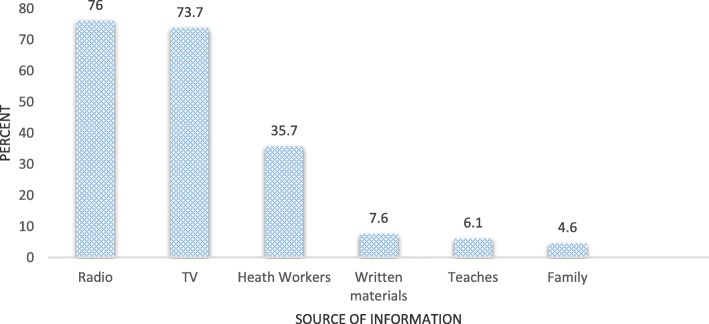
Source of information for women of 30–45 years about cervical cancer and screening in Jimma town, south west Ethiopia, June, 2017

About 524 (71.1%) and 484 (65.7%) of the respondents had ever heard about cervical cancer and cervical cancer screening test respectively. Among those ever heard about cervical cancer, only108 (20.6%) had good knowledge. Out of women who had good knowledge, 40.7% of them were undergone screening for cervical cancer (Fig. 1). The major source of information for the respondent about cervical cancer and screening was radio (Fig. 2).

### Constructs of the health belief model

About 265(50.4%) of respondents had positive perceived susceptibility towards cervical cancer. About three- fourth (75.3%) of respondents had positive perceived severity of cervical cancer. Three hundred eighty-seven (74.3%) of respondents had a positive perceived benefit towards cervical cancer screening. Three hundred eighty-eight (74.5%) had low perceived barriers for cervical cancer screening (Table 3).

**Table 3:** Constructs of HBM on study of cervical screening service utilization factors associated with cervical cancer screening service utilization among age-eligible women for cervical cancer screening in Jimma town, south west Ethiopia, June, 2017

Items	Strongly Disagree	Disagree	Neutral	Agree	Strongly Agree	Mean(±SD)
Perceived susceptibility						
You may get cervical cancer some time during your life	37	68	91	282	43	3.43(1.05)
It is likely that you will get cervical cancer in the future	57	164	31	178	91	3.16(1.33)
Your chances of getting cervical cancer in the next few years are high	49	122	79	204	67	3.23(1.21)
Perceived severity						
If you thought about cervical cancer you will worry	13	42	39	412	15	3.72(0.76)
When you think about cervical cancer, you will afraid	19	49	46	402	5	3.62(0.81)
Problems you would experience with cervical cancer would last a long time	8	201	75	233	4	3.05(0.96)
Cervical cancer would threaten a relationship with husband, or partner	7	133	44	270	67	3.49(1.05)
If you had cervical cancer your whole life would affected	11	35	34	303	138	4.00(0.89)
If you developed cervical cancer, you would not live longer than 5 years	11	58	71	265	116	3.80(0.98)
Perceived benefit						
Having cervical cancer screening regularly decrease worry of cervical cancer	7	111	31	248	124	3.71(1.09)
Having regular cervical cancer screening will help to find changes to cervix	4	110	237	169	1	3.10(0.75)
If cervical cancer was found at a cervical cancer screening its treatment would not be so bad	3	139	20	240	118	3.64(1.12)
Having a regular cervical cancer screening is the best to diagnosed early	7	104	350	54	6	2.89(0.59)
Having regular cervical cancer screening will decrease your chances of dying from cervical cancer	9	31	11	312	158	4.11(0.84)
Perceived barriers						
You may afraid to have a cervical cancer screening for fear of a bad result	72	292	28	87	42	2.49(1.16)
You don’t know where to go for a cervical cancer screening	109	303	42	54	13	2.15(0.95)
You would be ashamed to lie on a gynecologic examination table and show your private parts to have a cervical cancer screening	76	314	41	74	16	2.31(0.99)
Having a cervical cancer screening takes too much time	56	247	159	56	3	2.43(0.84)
Having a cervical cancer screening is too painful	44	235	171	55	16	2.55(0.90)
You neglect to have a cervical cancer screening regularly	35	245	82	91	68	2.83(1.19)
You have other problems more important than having a cervical cancer screening in your life	70	251	61	86	52	2.61(1.20)
You are too old to have a cervical cancer screening regularly	84	295	78	53	11	2.26(0.92)
There is no health center close to your house to have a cervical cancer screening	94	306	67	48	6	2.17(0.87)
You prefer a female doctor to conduct a cervical cancer screening	61	214	23	166	57	2.89(1.28)

### Magnitude of cervical cancer screening

**Fig. 3:**
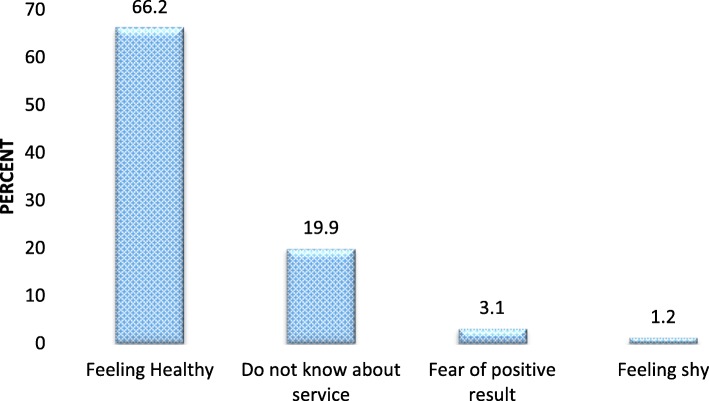
Reasons of decline for cervical cancer screening service among age-eligible women for cervical cancer screening in Jimma town, June, 2017

Among interviewed women, only about 114(15.5%) had a history of cervical cancer screening within the last 5 years. Three hundred fifty-three (48.9%) had a history of gynecologic examination for different reasons. Out of screened women, 63(55.3%) were screened because of personal initiative while the rest were by recommendation of health professionals. Two hundred thirty-eight (45.5%) of women have gender preference for gynecologic examination. From those who have gender preference for gynecologic examination, 209 (88.2%) prefer female physicians (health care providers). The most common reason for not being screened was feeling healthy (Fig. 3).

### Factors associated with cervical cancer screening

Variables like educational status, occupational status, knowing somebody with cervical cancer, knowing someone who screened for cervical cancer, history gynecologic examination, preferring gender of physicians for gynecological examination, advice from health care workers, knowledge status, perceived susceptibility, perceived severity, and perceived barrier were found to have a *p*-value less than 0.1 in bivariate analysis. In multivariable logistic regression, being government employee [AOR = 3.00, 95% CI: 1.49–6.01], knowing someone who has ever screened [AOR = 3.61, 95% CI: 2.07–6.29], having history of gynecologic examination [AOR =2.84, 95% CI: 1.48–5.45], not preferring gender of physician for gynecological examination [AOR = 3.57, 95% CI: 1.98–6.45], getting advice from health care providers [AOR = 4.45, 95% CI: 2.57–7.70], having good knowledge of cervical cancer screening [AOR = 3.46, 95% CI: 1.47–8.21] and having perceived susceptible for cervical cancer [AOR = 3.03, 95% CI: 1.64–5.56] were independently associated with cervical cancer screening (Table 4).

**Table 4:** Bivariate and Multivariable analysis of factors associated with cervical cancer screening service utilization among age-eligible women for cervical cancer screening in Jimma Town, south west Ethiopia, June, 2017

Variables	Screening status		Crude OR (95% CI)	AOR (95% CI
	Screened (%)	Not screened (%)		
Educational status				
No education	10(6.1)	156(93.9)	0.16(0.08–0.33)	0.68 (0.25–1.84)
Primary school	17(8.7)	179(91.3)	0.24(0.13–0.43)	0.49(0.31–1.75)
Secondary	26(16.3)	134(83.7)	0.49(0.29–0.82)	1.63(0.75–3.56)
College and above	61(28.4)	154(71.6)	1	
Occupational status				
Government employee	63(31.7)	136(68.3)	4.03(2.29–7.11)	3.00 (1.49–6.01)*
Daily laborer	5(6.5)	72(93.5)	1.46(0.73–2.87)	1.37 (0.36–5.18)
Merchants	27(13.1)	179(86.9)	1.28(0.44–3.69)	1.82(0.830–3.99)
House wife	19(7.5)	236(92.5)	1	1
Know somebody with cervical cancer				
Yes	82(33.3)	164(66.7)	3.81(2.42–6.00)	0.89(0.44–1.81)
No	32(11.6)	244(88.4)	1	1
Knowing someone who screened for cervical cancer				
Yes	82(40.5)	122(59.5)	6.00(3.79–9.52)	3.61 (2.07–6.29)*
No	32(10)	286(90)	1	1
History gynecologic examination				
Yes	93(26.3)	260(73.7)	2.54(1.52–4.25) 1	2.84(1.48–5.45)*
No	21(12.4)	149(87.6)		1
Preferring gender of physician for gynecological examination				
Yes	28(11.8)	210(88.2)	1	1
No	86(30.2)	199(69.7)	3.24(2.03–5.18)	3.57(1.98–6.45)*
Advice from health care worker				
Yes	76 (40.6)	111(59.4)	5.37(3.44–8.39)	4.45(2.57–7.70)*
No	38 (11.3)	298(88.7)	1	1
Knowledge status				
Good	57 (35.4)	104(64.6)	10.41(5.25–20.62)	3.47(1.47–8.21)*
Fair	46 (28.2)	117(71.8)	5.87(2.9–11.9)	2.82(1.31–6.10)*
Poor	11 (5.5)	189(94.5)	1	1
Perceived susceptibility				
High	89 (33.6)	176(66.4)	4.77(2.940–7.75)	3.02(1.64–5.56)*
Low	25(9.8)	236(91.2)	1	1
Perceived severity				
High	105(26.4)	289(73.6)	4.84(2.37–9.89)	2.30(0.97–5.48)
Low	9(7)	120(93)	1	1
Perceived benefit				
High	96(24.8)	291(75.2)	2.13(1.23–3.68)	1.13(0.55–2.34)
Low	18(13.4)	116(86.6)	1	1
Perceived barrier				
Low	20(15)	113(85)	1.81(1.06–3.07)	0.79(0.38–1.63)
High	94(24.2)	294(75.8)	1	1

* *p* < 0.05, *OR* odds ratio, *AOR* adjusted odds ratio

## Discussion

The aim of this study was to identify the factors associated with cervical cancer screening in Jimma, Ethiopia. This study showed that only 15.5% of surveyed women had reported a history of cervical cancer screening within the last 5 years. The finding was similar to the study conducted in Mekele Zone Northern Ethiopia, which shows cervical cancer screening uptake was 19.8% [13]. The similarity might be because in both studies, the study subjects were urban women who have access to media and other information. If women have information concerning cervical cancer severity, they may utilize screening services. But, this figure was higher when compared to the magnitude of cervical cancer screening at country-level in 2008 which showed only 1% of eligible women undergone screening in Ethiopia [9]. This discrepancy might be due to the study conducted at country in 2008 is late and currently cervical cancer screening service is one of routine health services for eligible women in Ethiopia. Moreover, in recent times different activities had been undertaken to increase the utilization of cervical cancer screening in Jimma town. For instance, there were campaigns prepared by health facilities providing screening services in the town and dissemination of information about cervical cancer through radio programs by Jimma University Medical Center. The finding was slightly lower than a community-based cross-sectional study conducted in Tanzania which was about 22% of study participants were undergone screening [27]. This discrepancy could be due to a difference in the social-demographic characteristics of the participants of the studies, and a study conducted in Tanzania included women between age of 18 and 49.

Occupational status was an independent predictor of cervical cancer screening service utilization. Government employee women were more likely to be screened when compared to housewives. This finding was similar to the study conducted in Nigeria, which shows employed women utilize cervical cancer screening service more likely than unemployed women [28]. This might be because the majority of employed women have higher educational status and they have access to information about cervical cancer and screening service from different sources. Also, this study showed that knowing someone who was ever screened for cervical cancer was associated with cervical cancer screening service utilization. Women who know someone ever screened were more likely to undergo cervical cancer screening when compared with women who do not know someone screened for cervical cancer. This result was consistent with the study done in Uganda [29]. This might be due to screened women discuss with unscreened women about the screening service, procedures, and the time it takes which decreases fear of women towards undergoing screening.

History of the gynecologic examination was also associated with the utilization of cervical cancer screening. Women who had the previous history of gynecologic examination for any reason were more likely to be screened for cervical cancer. This association can be explained by women who had a history of exposure for health professionals do not afraid to expose their genitalia for cervical cancer screening as they were familiarized on the previous examination. In addition to this, the previous examination may enforce them for cervical cancer screening.

In addition, women who do not prefer the gender of physicians for gynecological examination were more likely to undergo cervical cancer screening services. This finding is in line with the study conducted in Serbia [30]. This might be because of women who prefer the gender of physician for gynecologic examination miss the service when the service is provided by gender that they do not prefer. In contrast, women who do not have a gender preference for gynecologic examination will use the service whoever providing the service.

Consultation/advice from health professions was associated with cervical cancer screening service utilization. Women who had advice from health care providers were more likely to be screened when compared with women who had no advice. This finding is consistent with a study conducted in Jamaica and Uganda [29, 31]. This may be due to the information from health care providers increase awareness about the disease and the advantages of having screening services.

Furthermore, knowledge of cervical cancer and screening service determine cervical cancer screening utilization. This result was in line with a study conducted in Mekele Zone, Northern Ethiopia, Tanzania and Malawi which showed that women who had good knowledge about cervical cancer were more likely to utilize cervical cancer screening than those who have poor knowledge about cervical cancer and screening [13, 27, 32]. This may be due to knowledge about cervical cancer clear rumors about cervical cancer and increase their awareness about the advantage of undergoing screening.

The study also revealed that perceived susceptibility for cervical cancer was a factor that affect cervical cancer screening utilization. Women who had high perceived susceptibility were more likely to undergo screening when compared with low perceived susceptibility. This finding was in line with the study conducted in Mekele Zone, Northern Ethiopia, Kenya, and Thailand [13, 33, 34]. This may be because these women have information about the disease and know their susceptibility as the result they undergo screening to protect themselves.

### Limitations and strengths of the study

Since data were collected through self-reported questions, social desirability bias may affect the finding. Further, it could be difficult to identify whether outcome or predictor variables come first due to the nature of the cross-sectional study design. This is because data collection happen at a time for predictors and outcome variables. However, the study has some strengths. This study used the health belief model which has contributed to assessing behaviors. Further, we controlled the analysis for potential confounder variables.

## Conclusions and recommendations

Predictors of cervical cancer screening utilization were occupation of respondent, knowing someone who screened for cervical cancer, history of gynecologic examination, not preferring gender for gynecologic examination, consultation/advice about cervical cancer by health care providers, perceived susceptibility to cervical cancer and knowledge about cervical cancer. The prevalence of screening service is low in Jimma town. The common reasons for not undergoing screening were feeling of healthy. To increase cervical cancer screening utilization acting on knowledge and perceived susceptibility towards cervical cancer is crucial.

## Data Availability

All data generated during and/or analyzed during the study are available from the corresponding author on reasonable request.

## Abbreviations

AOR: Adjusted odds ratio
HIC: High income countries
HIV: Human immunodeficiency virus
HPV: Human papilloma virus
LMIC: Low and middle-income countries
VIA: Visual inspection of the cervix with acetic acid
